# Efficacy of Azatyrosine-Phenylbutyric Hydroxamides, a Histone Deacetylase Inhibitor, on Chemotherapy-Induced Gastrointestinal Mucositis

**DOI:** 10.3390/ijms20020249

**Published:** 2019-01-10

**Authors:** Po-Lin Liao, Shih-Hsuan Huang, Chien-Hung Hung, Wei-Kuang Huang, Chi-Hao Tsai, Jaw-Jou Kang, Hui-Po Wang, Yu-Wen Cheng

**Affiliations:** 1 Institute of Food Safety and Health Risk Assessment, School of Pharmaceutical Sciences, National Yang-Ming University, Taipei 11221, Taiwan; plliao@ym.edu.tw (P.-L.L.); jjkang@ntu.edu.tw (J.-J.K.); 2 School of Pharmacy, College of Pharmacy, Taipei Medical University, Taipei 11031, Taiwan; a001ou@gmail.com (S.-H.H.); garasaba@gmail.com (W.-K.H.); hpw@tmu.edu.tw (H.-P.W.); 3 Department of Pharmacology, School of Medicine, College of Medicine, Taipei Medical University, Taipei 11031, Taiwan; zaq8810@gmail.com; 4 Graduate Institute of Medical Sciences, College of Medicine, Taipei Medical University, Taipei 11031, Taiwan; 5 Institute of Toxicology, College of Medicine, National Taiwan University, Taipei 10051, Taiwan; d01447001@ntu.edu.tw

**Keywords:** anti-inflammation, azatyrosine-phenylbutyric hydroxamides (PBHA), histone deacetylase inhibitor (HDACi), mucositis

## Abstract

Gastrointestinal mucositis is a serious side effect of chemotherapy. Currently, no effective treatment exists for chemotherapy-induced mucositis, prompting the need to develop an anti-mucositis agent for use in clinics. The present study investigated whether azatyrosine-PBHA (AzP), a histone deacetylase inhibitor, has a therapeutic effect on intestinal mucosa. The results indicated that AzP did not affect the proliferation and viability of cancer cells, outcomes that are achieved by suberoylanilide hydroxamic acid (SAHA). However, AzP could decrease production of the inflammatory mediators interleukin-6 (IL-6), monocyte chemoattractant protein-1 (MCP-1), and tumor-necrosis factor-α (TNF-α). In vivo histopathological assessment showed that AzP reduced cisplatin-induced injury to the jejunum villi and triggered weight loss in the C57BL/6 mice. Immunohistochemistry (IHC) results demonstrated that mice treated with AzP also recovered from cisplatin-induced injury to the intestinal mucosa. Mechanistic in vitro study using DAVID/KEGG enrichment analysis of microarray data and confirmation by a Western blot indicated the influence of AzP on the MEK/ERK and AKT-dependent pathway. In conclusion, the study demonstrated that AzP might regulate the MEK/ERK MAPK signaling pathway to attenuate MCP-1, TNF-α, and IL-6 production and provide opportunities for the development of new anti-inflammatory drugs targeting mucositis.

## 1. Introduction

Gastrointestinal mucositis is a frequent and severe side effect of radiotherapy and chemotherapy in cancer patients [1,2]. Approximately 50 to 80% of patients suffered from mucositis, with the occurrence being dependent on the type of chemotherapy; vomiting, abdominal pain, and severe diarrhea [3] were the most common symptoms experienced. A life-threatening stage may also be seen in patients, and severe physical obstruction of food and water intake leads to weight loss. A septic complication is also a feature of this stage and occurs due to a loss in protective epithelial and basement membrane barriers [4]. Small intestinal mucositis leads to reductions in the drug dosage and delayed treatment compromises the effectiveness of chemotherapy [5]. This increases its likelihood of being the most limiting factor associated with cancer chemotherapy and radiotherapy. Much research has been conducted to elevate the quality of life among cancer-treated patients and 5 pathological stages of mucositis has been defined: initiation, primary damage response, signal amplification, ulceration and healing [5]. Cellular inflammatory response was found to occur during mucositis and increased levels of pro-inflammatory cytokines such as the tumor-necrosis factor-α (TNF-α) and interleukin-6 (IL-6) are consistently seen in the peripheral blood and/or mucosa of patients, further showing their pivotal roles in this inflammatory process. At present, cisplatin is widely prescribed in chemotherapy for a variety of human cancers. The clinical use of cisplatin is associated with side effects at the gastrointestinal level with development of gastrointestinal mucositis reducing its therapeutic efficacy and contributing to its withdrawal from use in a clinical setting [6].

**Figure 1:**
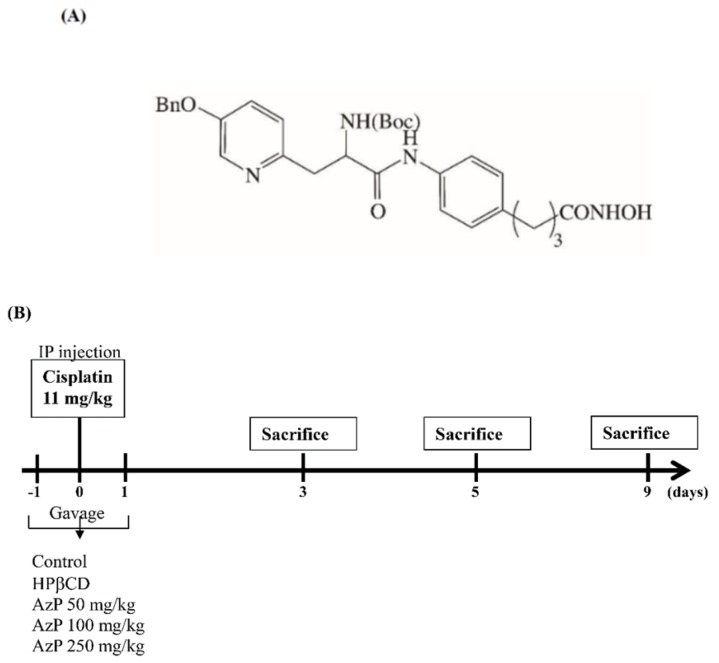
(**A**) Structure of Azatyrosine-phenylbutyric hydroxamides (PBHA), AzP; (**B**) A schematic of AzP on Cisplatin-induced mucositis. C57BL/6 mice were treated with 11 mg/kg/day cisplatin on day 0. Gavages of 50, 100, and 250 mg/kg AzP were given on days −1, 0, and 1. From a total of 9 mice, 3 were sacrificed on days 3 and 5, and their intestine collected for further immunohistological observations. Weights were recorded daily until day 9.

Histone acetylation modulated by histone deacetylases (HDACs) serves as a key regulatory mechanism for gene expression and is involved in numerous developmental processes and diseases [7]. Many histone deacetylase inhibitors (HDACi) previously developed, had multiple therapeutic purposes for cancer, chronic inflammatory disease, bone engineering, and neurodegenerative diseases [8]. Phenylbutyric acid (PBA), an FDA approved pan-histone deacetylase inhibitor drug, was originally used for the treatment of urea cycle disorders (UCDs) [9]. Various reports have found other biological activities in addition to the role of HDAC inhibition as an ammonia scavenger of PBA [10]. These include the induction of cell growth arrest, differentiation and apoptosis in several tumor cells [11]. Other studies have also reported that HDACs attenuate inflammation (e.g., mucositis induced by chemotherapy or radiotherapy) via suppressing the expression of pro-inflammatory cytokines [12,13,14,15]. Based on the structure of PBA, patented methods have been invented to provide a series of amino acid-linker-phenylbutyric hydroxamides compounds (AA-PBHAs) using positively charged amino acids as delivery moieties. These compounds direct PBA towards negatively charged mucus tissues during mucositis [5] (US Patent Grant US 8242282 B2) with azatyrosine-PBHA (AzP, Figure 1A) presenting the strongest HDAC inhibition activity. 

There are very limited therapeutic agents available for mucositis treatment [16], and an N-truncated recombinant human keratinocyte growth factor-1, palifermin (Kepivance™), was the first agent approved for treating mucositis. This drug exhibits its effects by inhibiting apoptosis and DNA damage of epithelial cells, reducing pro-inflammatory cytokines expression, as well as promoting migration, proliferation and differentiation of damaged epithelial cells [17,18]. The major concern of palifermin is the potential oncogenic effect, which would compromise cancer treatment and the cost associated [19,20]. Therefore, the objective of this study was to investigate the therapeutic potential of AzP on mucositis.

## 2. Results

### 2.1. Protective Effect of Azatyrosine-Phenylbutyric Hydroxamides (Aza-PBHA) in Mucositis Model

**Figure 2:**
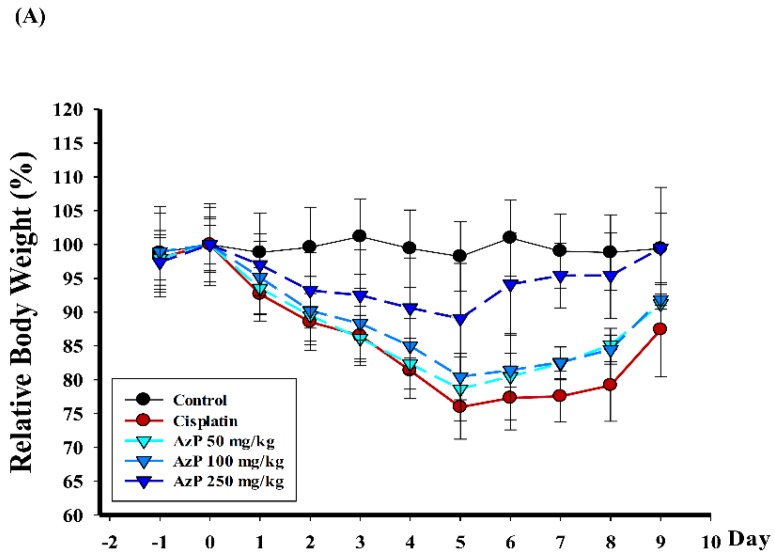

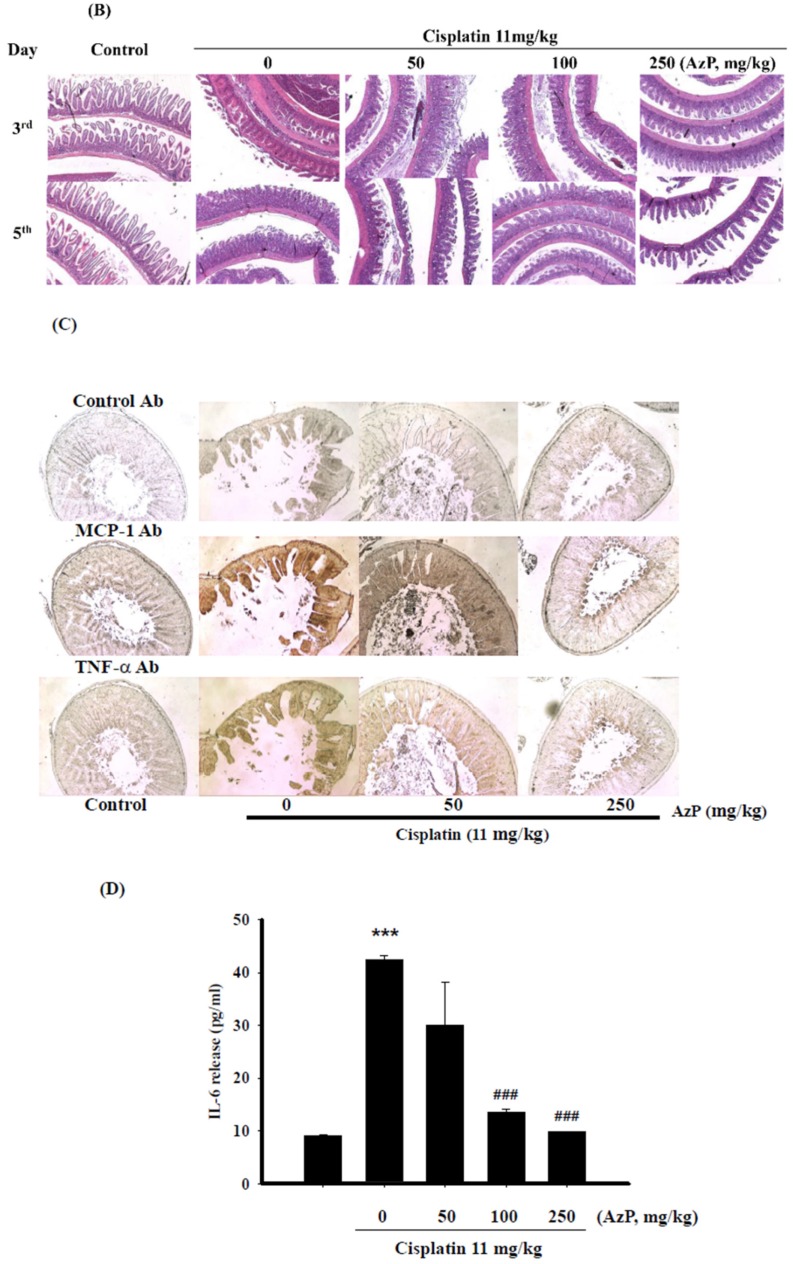
Efficacy of AzP in cisplatin-induced mucositis model of C57BL/6. (**A**) Weights recorded from C57BL/6 mice treated with 11 mg/kg cisplatin only with gavage of hydroxypropyl-β-cyclodextrin (HPβCD) and 50, 100, and 250 mg/kg/day AzP from days −1 to 9 are shown. The changes in weight were analyzed by calculation of the percentage of change from baseline. Relative body weight change (%) is shown with the data expressed as mean ± standard deviation (SD).; (**B**) The intestinal mucosa of C57BL/6 mice experienced thinning, a consequence of cisplatin chemotherapy. Mice pretreated with AzP for 3 days had thickened mucosa relative to chemotherapy-treated controls. This was the result of longer villi and deeper crypts; however, a similarity to normal control from the swiss-roll trimming on days 3 and 5 was observed. Images are at 40× magnification; (**C**) Effect of AzP on cisplatin-induced inflammatory protein expression in jejunum from C57BL/6 mice on day 5. Mice received AzP dosages of 0, 50, and 250 mg/kg, 30 min before i.p. injection with 11 mg/kg cisplatin to induce mucositis. After 120 h of stimulation, jejunum sections were analyzed using the immunohistochemistry (IHC) method. Images are at 100× magnification; (**D**) Serum IL-6 level was detected on day 5. Data expressed as mean ± SD. *** *p* < 0.001 compared with control and ### *p* < 0.001 compared with AzP-treated group.

In this study, hydroxypropyl-β-cyclodextrin (HPβCD) was used as an excipient with AzP, and the ratio 1:10 (AzP: HPβCD, *w*/*w*) was selected for further research. Gavages of 50, 100, and 250 mg/kg of AzP were performed on days −1, 0 and 1, while cisplatin (11 mg/kg) was administered via intraperitoneal (IP) injection to C57BL/6 mice on day 0. The control mice were given PO HPβCD via sham-injection. No mortality was observed over the study period; however, cisplatin induced weakness in all animals within the cisplatin treatment group. This was followed by weight reduction throughout the experimental period. Relative mean body weight change (%) was recorded daily and expressed as mean ± standard deviation(SD) from baseline (day 0 = 100%). At day 5, 11 mg/kg cisplatin-only treatment reduced body weight to 76.0 ± 4.7%, while administering with AzP 50 mg/kg, 100 mg/kg, and 250 mg/kg at day 5 rescued the AzP-reduced weight loss to 78.7 ± 4.7, 80.5 ± 3.4 and 89.1 ± 8.1%, respectively (Figure 2A). Intestine samples were collected from C57BL/6 mice on days 3 and 5 and were used to perform a swiss-rolled trimming for H&E staining; representative photographs are shown in Figure 2B. Structural alterations in the small intestinal mucosa and damage were observed in the cisplatin-only treatment group on days 3 and 5. Pretreatment with AzP showed the potential to rescue cisplatin-induced injury, especially in the 250 mg/kg/day group, as structure of intestinal mucosa, villus length and crypt depth were restored, appearing similar to the control group (Figure 2B). In addition, to investigate the anti-inflammatory efficacy of AzP, the inflammatory cytokines monocyte chemoattractant protein-1 (MCP-1) and TNF-α were stained using intestine samples collected on day 5. Results showed that cisplatin-only treatment significantly increased the expressions of MCP-1 and TNF-α, while AzP dose-dependently reduced these expressions (Figure 2C). Systematic inflammation was evaluated using ELISA targeting IL-6; AzP was proven to inhibit cisplatin-induced IL-6 production in a dose-dependent manner (Figure 2D). 

### 2.2. Aza-PBHA Showed Histone Deacetylase (HDAC) Inhibition Activity

To further investigate the mechanism underlying AzP-possessed anti-inflammatory responses, the effects of AzP on HDAC activity were examined. Pan HDAC, HDAC1, HDAC 3, and HDAC 4 activity were tested using a commercial fluorescence assay, and suberoylanilide hydroxamic acid (SAHA; Zolinza, Whitehouse Station, NJ, USA), the best characterized inhibitor, was used for comparison. 

**Figure 3:**
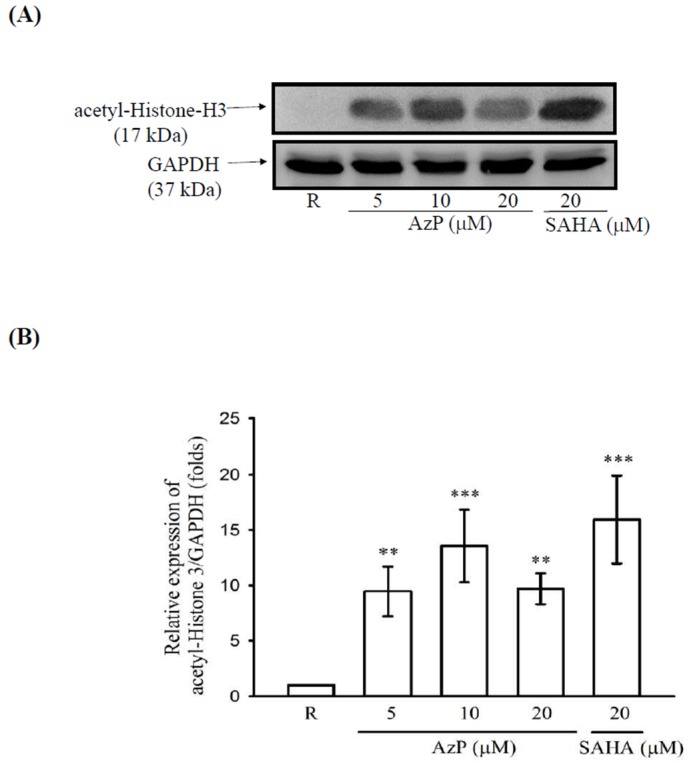
In vitro histone deacetylase (HDAC) activity. (**A**) The epithelial intestinal cell line IEC-6 cells (1 × 10^6^ cells/mL) were dispensed on 6 cm dishes and treated with the indicated concentrations of AzP (5, 10 and 20 μM) or suberoylanilide hydroxamic acid (SAHA) (20 μM) for 24 h. Cell lysates were used for acetyl-histone H3 protein expression by Western blot. Data are expressed as means ± SD from 3 independent experiments. *** *p* < 0.001 as compared with the resting and ** *p* < 0.01 as compared with the resting (**B**).

As shown in Table 1, the results showed that IC_50_ of pan-HDAC, HDAC1, HDAC 3, and HDAC 4 of AzP were 1650 ± 103 nM, 3440 ± 680 nM, 660 ± 99 nM and 1970 ± 110 nM, respectively. Results from Western blot also confirm that AzP elevated histone acetylation (Figure 3A,B). 

**Figure 4:**
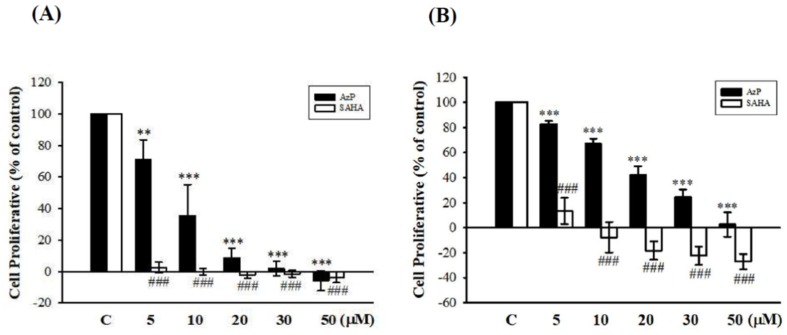
Effects of AzP and SAHA on A549 and Met-5A cell proliferation. A549 (**A**) and Met-5A (**B**) cells (2 × 10^4^) were dispensed on 96-well plates, SRB assay was performed treated with the indicated concentrations of AzP (5, 10, 20, 30, and 50 μM) and SAHA (5, 10, 20, 30, and 50 μM) for 48 h. Cell Proliferation % = {(T48 − T0)/T0} × 100. T48: the signal measured after 48-h treatment, T0: the signal measured before treatment at time 0. Data are expressed as means ± SD from 4 independent experiments, while a minus value indicates cell death. ### *p* < 0.001 as compared with the SAHA control, ** *p* < 0.01 as compared with the AzP control and *** *p* < 0.001 as compared with the AzP control.

The cancer cell line, A549, and the normal cell line, Met-5A, were used to compare the effects between AzP and SAHA on cell proliferation using SRB assay. AzP concentration-dependently decreased cell viability of the A549 cancer cell line (5 μM: 70.82%, 10 μM: 35.48%, 20 μM: 8.48%, 30 μM: 1.79%, 50 μM: −5.83%) while SAHA exhibited more potency in decreasing cell viability (5 μM: 2.41%, 10 μM: −0.15%, 20 μM: −2.14%, 30 μM: −1.54%, 50 μM: −3.60%, Figure 4A), suggesting that AzP inhibited cell growth while SAHA resulted in significant cell death in A549 cells. In the normal Met-5A cell line, AzP concentration-dependently reduced cell viability (5 μM: 82.67%, 10 μM: 67.05%, 20 μM: 41.97%, 30 μM: 24.43%, 50 μM: 2.59%), while SAHA caused significant cell death (5 μM: 13.42%, 10 μM: −7.67%, 20 μM: −18.32%, 30 μM: −22.44%, 50 μM: −27.18%, Figure 4B).

### 2.3. AzP Exerted In Vitro Anti-Inflammatory Potential

**Figure 5:**
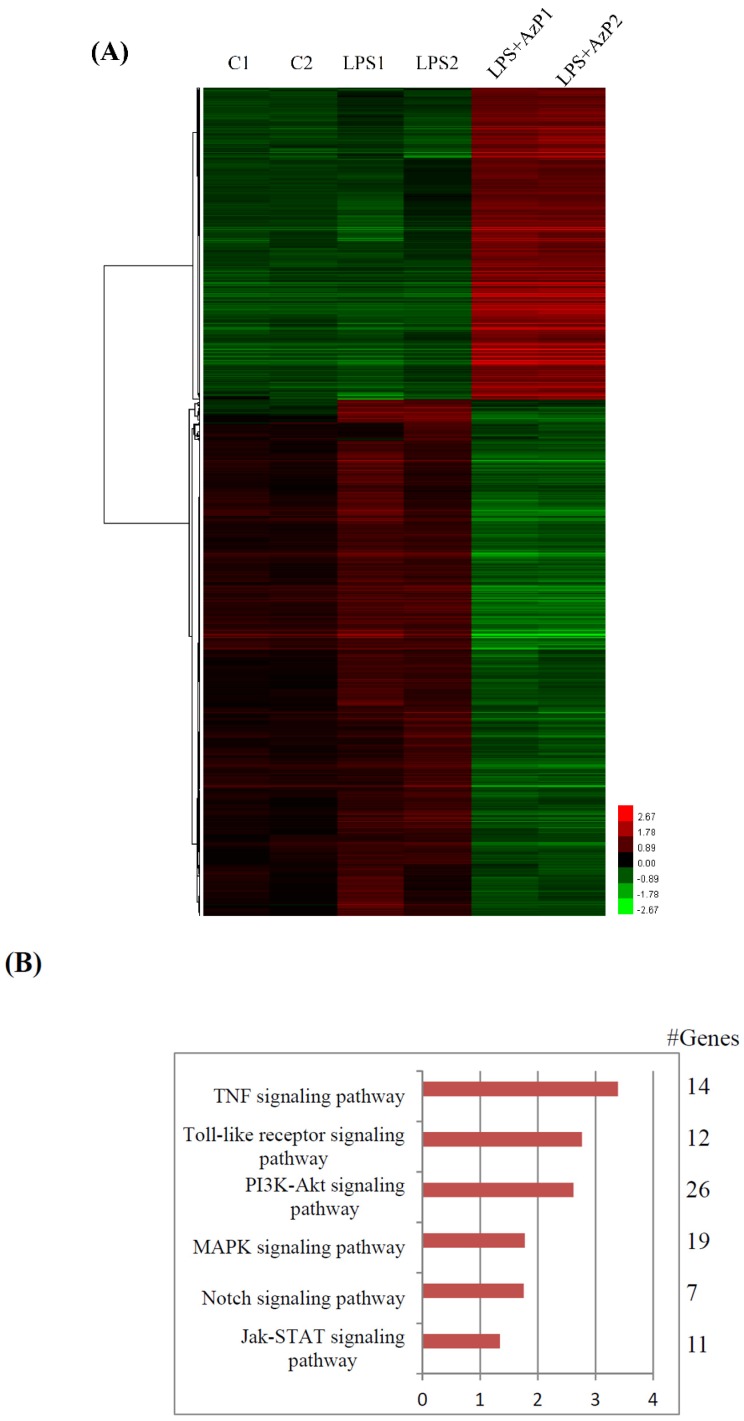

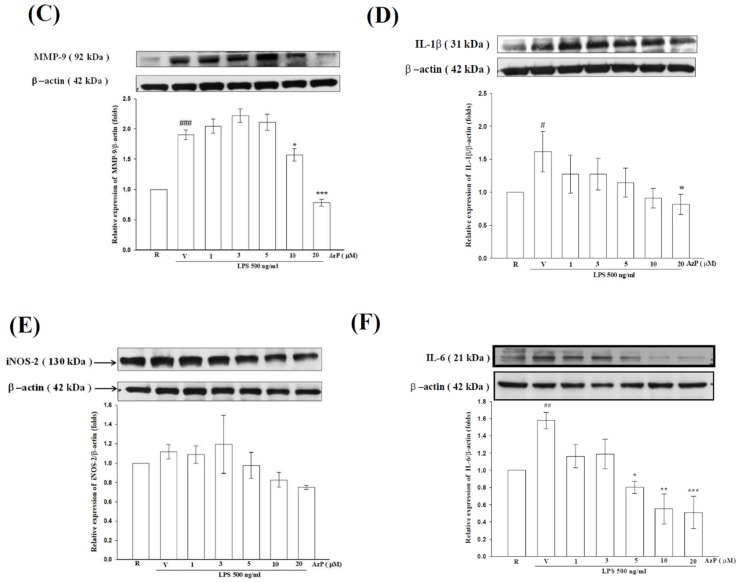
Hierarchical clustering and KEGG analyses of gene profiles of the AzP-manipulated IEC-6 cells. (**A**) Two independent experiments were performed, samples were named C1, C2 as control group (1% dimethyl sulfoxide (DMSO)); LPS1, LPS2 as lipopolysaccharide (LPS) 500 ng/mL group; LPS + AzP1, LPS + AzP2 as LPS 500 ng/mL and AzP 20 μM group. Heat maps showing the clustering analysis of the IEC-6 cells with different treatments (1% DMSO, LPS 500 ng/mL and/or AzP 20 μM) indicates up-regulation (red), down-regulation (green), and mean gene expression (black). (**B**) DAVID KEGG pathway analysis of these genes indicates that several pathways were significantly involved in the response (*p* > 10 of −log10 value). IEC-6 cells were treated with LPS 500 ng/mL for 8 h and/or AzP 1–20 μM, Western blot performed and immunoblotted with several inflammatory protein expressions including MMP-9 (**C**), IL-1β (**D**), iNOS (**E**), and IL-6 (**F**). Density was measured and calculated. ### *p* < 0.001 as compared with the control, ## *p* < 0.01 as compared with the control, # *p* < 0.05 as compared with the control, * *p* < 0.05 as compared with the LPS-treated group, ** *p* < 0.01 as compared with the LPS-treated group and *** *p* < 0.001 as compared with LPS-treated group.

To confirm the therapeutic efficacy of AzP in C57BL/6 mice, duplications of the small intestinal epithelial cell line, IEC-6, cells were treated with 1% dimethyl sulfoxide (DMSO) (C1, C2), lipopolysaccharide (LPS, 500 ng/mL) (LPS1, LPS2), or AzP (20 μM) co-treated with LPS (500 ng/mL) (LPS + AzP1, LPS + AzP2) for 8 h. Microarray using ROA v2.1 Rat OneArray^®^ (Phalanx Biotech Group, Inc., Hsinchu, Taiwan) was then performed twice in terms of the technical replicates, with a reproducibility of more than 0.975. Criteria of identification of differentially expressed genes were: fold change ≥2 or ≤ 2 and *p*-value < 0.05. Results showed that 2420 and 74 of 20,715 genes were significantly affected by AzP/DMSO and LPS/DMSO treatment, respectively. A total of 1159 and 25 descending genes and 1261 and 49 ascending genes were identified from AzP/DMSO and LPS/DMSO comparison, respectively. The heat maps of up-regulation (red), down-regulation (green), and mean gene expression (black) of 836 inverse correlation genes between two comparisons (AzP/LPS vs. LPS/Control) followed by hierarchical clustering analysis are presented in Figure 5A. Detailed individual genes are shown in Supplementary Figure S1. Pathway analysis using KEGG indicated that six pathways may be involved, including NOD-like receptor signaling pathway (Figure 5B). The inhibitory effect of AzP on LPS-induced inflammatory protein expression was further confirmed by treating IEC-6 cells with 500 ng/mL LPS for 8 h. Co-treating with AzP (1–20 μM) concentration-dependently inhibited LPS-induced MMP9, IL-1β, and IL-6 protein expressions (Figure 5C,D,F, respectively).

### 2.4. Possible Molecular Mechanisms of AzP in IEC-6 Cells

**Figure 6:**
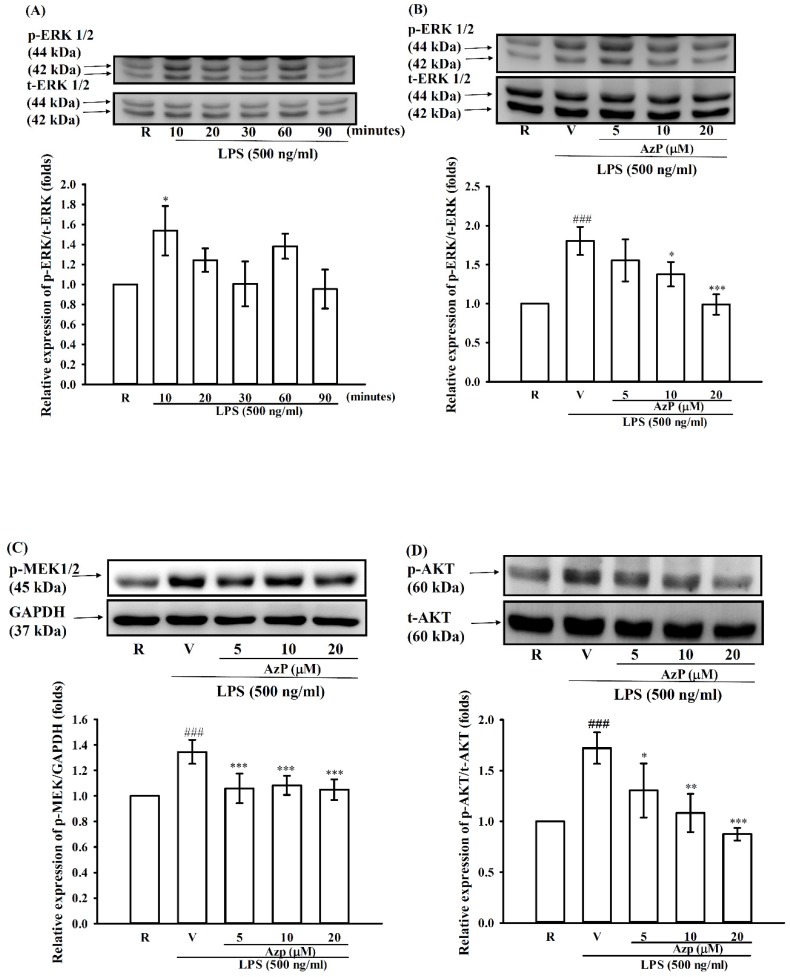
Western blotting of IEC-6 cells treated with LPS and/or AzP. (**A**) IEC-6 cells (1 × 10^6^ cells/mL) were dispensed on 6 cm dishes and (**A**) treated with LPS (500 ng/mL) for 10, 20, 30, 60, and 90 min. Data expressed as means ± SD from 3 independent experiments. * *p* < 0.05 as compared with the resting. (**B**) Treatment with LPS (500 ng/mL) for 10 min as indicated. Cells were treated with the indicated concentrations of AzP (5, 10, and 20 μM) or vehicle for 30 min before treatment with LPS. Cell lysates were collected for Western blot targeting p-ERK phosphorylation (Thr202/Tyr204, **B**), MEK phosphorylation (Ser271/221, **C**), and Akt phosphorylation (Ser473, **D**). Data expressed as means ± SD from 3 independent experiments. ### *p* < 0.001 as compared with the resting, * *p* < 0.05, ** *p* < 0.01 and *** *p* < 0.001 as compared with the vehicle.

To further investigate the possible mechanism underlying the anti-inflammatory effect of AzP, phosphorylation of MAPK proteins and Akt was tested in IEC-6 cells stimulated with 500 ng/mL LPS and/or 5, 10, and 20 μM AzP treatment. LPS significantly induced ERK phosphorylation up to 1.8 ± 0.3-fold compared to the control at 10 min (*p* < 0.5, Figure 6A). 5–20 μM AzP were able to dose-dependently reverse LPS-induced ERK activation (Figure 6B). Furthermore, we found that IEC-6 cells treated with LPS for 10 min also significantly elevated MEK1/2 and Akt phosphorylation, while 5–20 μM AzP concentration-dependently reversed these inductions. This indicates a possible role in AzP-excreted anti-inflammatory effect (Figure 6C,D). 

### 2.5. AzP Did Not Cause Mutagenicity or Clastogenicity In Vitro or In Vivo

To test whether AzP possesses potential genotoxicity, the Ames test, chromosome aberration test, and micronucleus test were performed. No mutagenicity or clastogenicity were found (Table 2). 

In Ames tests, the number of revertants did not increase after treatment with various concentrations of AzP with or without S9 metabolic activation in all 5 test strains (TA1537, TA1535, TA98, TA100, and TA102; Supplementary Table S1). 

AzP concentrations of 0, 5, 10, and 20 μg/mL resulted in no abnormal increase in damaged chromosomes (Supplementary Table S2). For the micronucleus assay, the ratio of PECs to total erythrocytes (%) and the frequencies of MNPCEs in the treatment groups (5, 50, and 500 mg/kg body weight) showed no significant differences compared to the negative control group treated with the same amount of vehicle (distilled water; Supplementary Table S3). 

## 3. Discussion 

Many reports have indicated that patients undergoing chemotherapy would often experience the side effect of mucositis, with the pro-inflammatory cytokines IL-6 and TNF-α playing a key role in chemotherapy-induced mucositis [21,22]. Drugs for treating chemotherapy without associated mucus damage have long been an unmet clinical need. Much research has used HDAC inhibitors as anti-cancer drug treatments, and the anti-inflammatory therapeutic potential of HDAC inhibitors has recently been discovered [8]. A series of azatyrosine-derived hydroxamates, originally designed as histone deacetylase inhibitors, were proven to exhibit anti-inflammatory activities (USA Patent No. 8,242,282 B2). One of the analogues, AzP, was selected for detailed investigation in chemotherapy-induced mucositis and its possible underlying mechanism of action examined. 

Cisplatin, commonly used for testicular cancer, ovarian cancer and head and neck cancer treatments, causes mucositis and its effects on mucous membranes have been reported [23]. A concentration of 11 mg/kg of cisplatin by intraperitoneal injection was administered to C57BL/6 mice on the 5th day of study; weight loss (Figure 2A), increased inflammatory cytokine expression of MCP-1 and TNF-α in the corresponding jejunum (Figure 2C) and IL-6 from serum expression (Figure 2D) were observed. The shortening and lysing of villi were also observed on the 3rd and 5th day in the cisplatin-treated animals (Figure 2B), indicating that 11 mg/kg of cisplatin resulted in damage to the epithelial cells, wrecking of the barrier in jejunum and releasing relative inflammatory cytokines. Consecutive gavage of AzP for 3 days (−1, 0 and 1 day) proved effective in rescuing cisplatin-damaged structures (Figure 2).

HDAC inhibition abilities were compared between AzP and SAHA, a well-known anti-tumor HDACi, by triggering autophagy and apoptosis [24]. SAHA presented stronger HDAC inhibition ability with a pan-HDAC inhibitory IC_50_ of 84.5 ± 13.7 nM, while IC_50_ of AzP was 1650 ± 103 nM (Table 1). AzP reduced cell growth in cancer cell line, A549, and normal cell line, Met-5A, while SAHA caused significant cell death in both cell lines (Figure 4); SAHA showed more toxicity than AzP. Interestingly, cisplatin reduced IEC-6 viability while AzP did not influence cisplatin-reduced cell viability (data not shown); the protective role of AzP among chemotherapy-induced mucositis does not occur via rescuing cisplatin-reduced cell viability. 

To further elucidate the possible modes of action, complete mRNA expression was screened using microarray analysis in IEC-6 cells stimulated with LPS 500 ng/mL and/or 20 μM AzP using DAVID/KEGG enrichment analysis of microarray data (*n* = 2) (Figure 5B). The most related inflammatory cytokine expression was confirmed using Western blot, which included MMP-9, IL1β, and IL-6 proteins; the protein expression of iNOS was unaffected (Figure 5C). Mitogen-activated protein kinases (MAPKs), including ERK, p38, and JNK, were reported to be involved in toll-like receptor-mediated inflammatory responses [25,26]. LPS induced phosphorylation of MAPKs, while AzP effectively reduced ERK phosphorylation (Figure 6A); p38 and JNK were unaffected by AzP (data not shown). Upstream of ERK, MEK phosphorylation was significantly restored by AzP treatment compared with the LPS-only group, indicating the participation of the MEK/ERK pathway in AzP-caused efficacy. In addition, the PI3K-AKT pathway was involved in cell proliferation, survival and cytoskeleton restoration. Recent reports showed that HDAC inhibitors may act as an Akt inhibitor to further activate FOXO1 followed by proliferation inhibition [27]. Our results showed that AzP strongly reduced LPS-activated Akt, showing that AzP may inhibit proliferation through Akt inhibition. Furthermore, results from the microarray assay showed that AzP could reduce LPS-induced p65 mRNA expression. Results from Western blot of both p65 phosphorylation and its upstream IKK phosphorylation were; however, unaffected; thus, further analysis is required to confirm the role of NF-kb among MOA of AzP.

To conclude, AzP showed strong inhibitory effect on chemotherapy-induced mucositis, possibly through MEK/ERK and/or PI3K/Akt pathway. Combined with the negative results from genotoxicity evaluation, we believe AzP possesses high therapeutic potential as a safe and strong anti-inflammatory agent for mucositis treatment.

## 4. Materials and Methods

### 4.1. Chemicals and Reagents

Azatyrosine-PBHA (AzP, Figure 1A) was synthesized using a patented method (US Patent Grant US 8242282 B2). The dry powder was collected and stored at 4 °C. In in vitro models, AzP was readily dissolved in DMSO; while in the in vivo models, AzP was dissolved with hydroxypropyl beta-cyclodextrin (HPβCD), a sugar derivative of good solubilizing capacity, in a 1:10 ratio.

Fetal bovine serum (FBS), Dulbecco’s modified Eagle’s medium (DMEM), Ham’s F-12K medium, penicillin/streptomycin/glutamate and trypsin-EDTA were obtained from Gibco BRL (Grand Island, NY, USA). The horseradish peroxidase (HRP)-conjugated anti-mouse immunoglobulin G (IgG) Ab and HRP-conjugated anti-rabbit IgG Ab were obtained from Amersham Biosciences (Sunnyrale, CA, USA). Except for acetyl-Histone H3 (Millipore, San Diego, CA, USA), other primary antibodies were purchased from Cell Signaling Technology (Beverly, MA, USA). All of the other chemical reagents were purchased from Sigma-Aldrich (St. Louis, MO, USA).

### 4.2. Cell Culture 

Rat intestinal epithelial cell line, IEC-6, and Chinese hamster ovary epithelial cells (CHO-K1) were obtained from American Type Culture Collection (Manassas, VA, USA). IEC-6 cells were cultured in DMEM high glucose medium (4.5 g/L) with 10% heat-inactivated fetal bovine serum (FBS) while CHO-K1 cells were maintained in F12K high glucose medium (4.5 g/L) supplemented with 10% heat-inactivated fetal bovine serum (FBS) in a humidified atmosphere of 5% CO_2_ at 37 °C.

### 4.3. HDAC Enzyme Activity Assay 

Pan HDAC enzyme (all the known class I HDACs-HDAC1, HDAC2, HDAC3, HDAC8 and class II HDACs 4-7, 9 and 10), HDAC subtype 1, 3 and 4 activity were determined by using the HDAC fluorometric cellular activity assay (Enzo Life Sciences, Farmingdale, NY, USA) according to the manufacturer’s protocol. Different concentrations of AzP and SAHA were used, and the fluorescence intensity was measured on a fluorometric reader using excitation/emission wavelength of 360/460. Percent activity for each of the test compounds is calculated as follows: 

Activity = {(Sc − B)/(S° − B)} × 100. Wherein, S c represents the signal measured in the presence of AzP or SAHA, S° denotes signal measured in the absence of AzP or SAHA, B is measured in blank wells containing the medium alone. IC_50_ corresponds to the drug concentration which achieves 50% activity of the untreated control. IC_50_ was then calculated and represented as mean ± SD.

### 4.4. Animal Housing

Following acclimatization, 25 male ICR mice (6-week-old upon receipt, BioLASCO Ltd., Taipei, Taiwan) were randomly placed in 5 cages for micronucleus testing lasting 1 week. In addition, 45 female C57BL/6 mice (8-week-old upon receipt, BioLASCO Ltd., Taipei, Taiwan) were used as mucositis models. All the animals were kept under a 12 h light/dark cycle at 23 ± 1 °C and 39–43% relative humidity. All animals had free access to water and food (Rodent LabDiet 5001; PMI Nutrition International, LLC, Richmond, IN, USA). All experimental procedures followed the “Guide for the Care and Use of Laboratory Animals” (National Academy of Sciences Press, 1996). The experimental designs were reviewed and approved by the Institutional Animal Care and Use Committee of Taipei Medical University (approval number: LAC-2013-0037, 21 May 2013).

### 4.5. Mucositis Model

For the mucositis model experiments using chemotherapy, 45 female C57BL/6 mice were randomly placed in 5 groups for 3 days of treatment with AzP (50–250 mg/kg) P.O. on days −1, 0 and 1. Cisplatin (11 mg/kg) was intraperitoneally injected on day 0 as the chemotherapy. Control mice were injected with an appropriate vehicle. Mice were weighed daily and expressed as relative mean body weight change (%) from baseline (day 0 = 100%) and monitored closely for signs of morbidity over the course of the weeks to follow. On days 3 and 5, 3 of 9 mice were randomly chosen from each group and sacrificed for further intestinal analysis (Figure 1B). Control mice were injected with HPβCD as vehicle. 

### 4.6. Histological and Immunohistological Observations of Crypt and Villus

Histological and immunohistological observations of the intestine were performed on mice pretreated with AzP or HPβCD, followed by a course of cisplatin chemotherapy as described above. The mice were euthanized, intestines were removed, and a segment of the swiss-rolled trimming jejunum underwent formalin fixation and paraffin embedding. Hematoxylin and eosin stain were performed to observe the morphometry of villus length and crypt depth [28]. Also, tissue sections were deparaffinized and rehydrated through a series of alcohols followed by antigen retrieval with Tris-EDTA buffer (10 mM TrisBase, 1 mM EDTA, 0.05% Tween 20, pH 9.0). Then, the HRP/DAB (ABC) Detection immunohistochemistry (IHC) Kit (Abcam, Cambridge, UK) was used. Tissue sections were quenched with endogenous peroxidase (3% hydrogen peroxide) for 10 min, followed by 5% Bovine serum albumin (BSA) blocking for 5 min and subsequent incubation with primary antibodies overnight at 4 °C. Following washing, the tissue sections were incubated with biotinylated secondary antibody for 10 min, followed by streptavidin peroxidase for another 10 min. After washing, the colorimetric reaction was developed by incubation with DAB substrate for 5 min and underwent microscopy observation. 

### 4.7. Western Blot Analysis

IEC-6 cells were incubated in different AzP concentrations with/without LPS stimulation for 24 h. The homogenate from cells or tissues was then centrifuged at 14,000× *g* for 10 min at 4 °C and the supernatant was collected. Protein separation was performed using 10% reducing SDS-PAGE, electrotransferred onto polyvinylidene difluoride (PVDF), and immunoblotted with antibodies against inflammatory proteins as well as β-actin as control. Densitometry was performed using BioLight software V2000.01 (Uhldingen-Mühlhofen, Germany). The optical density of specific protein was normalized to an internal control and expressed as a ratio to control.

### 4.8. Oligonucleotide DNA Microarray 

IEC-6 cells were treated with 1% DMSO, lipopolysaccharide (LPS, 500 ng/mL), or AzP (20 μM) for 8 h, and samples from an independent duplication were collected for DNA microarray. Total RNA was extracted from cells using TRIzol reagent (Invitrogen, Carlsbad, CA, USA). The RNA concentration and purity were checked using OD_260/280_ values (>1.8) and OD_260/230_ values (>1.6), and the yield and quality were assessed using an Agilent 2100 Bioanalyzer (Agilent Technologies, Santa Clara, CA, USA). The ROA v2.1 Rat OneArray^®^ (Phalanx Biotech Group, Inc., Hsinchu, Taiwan) was used to assess gene expression with a total of 20,715 probes corresponding to the annotated genes in RefSeq v65 and Ensembl v76 databases, and 485 control probes used for the purposes of the study. The signal intensities were loaded into Rosetta Resolver^®^ System (Rosetta Resolver V7.2, Kenilworth, NJ, USA) to do data preprocessing and 75 percentile centering normalization was applied. The errors of the sample were estimated by using the error-weighted approach at the same time. Both fold change and *p*-value for pair-wise sample comparison were calculated to evaluate differentially expressed genes, which was followed by further KEGG enrichment analysis (Kyoto Encyclopedia of Genes and Genomes, https://www.genome.jp/kegg/). Using the DAVID v6.8 system (the Database for Annotation, Visualization and Integrated Discovery, https://david.ncifcrf.gov/), unsupervised hierarchical clustering by was performed by average linkage algorithm on selected differentially expressed gene lists after data transformation and mean centering.

### 4.9. Salmonella/Microsome Reversion Assay: Ames Test

Five strains of *Salmonella typhimurium*, TA98, TA102, TA100, TA1537, and TA1535, were obtained from Moltox (Boone, NC, USA). Mutagenicity was evaluated by the incorporation method in the presence or absence of S9 metabolic activation [29]. A volume of 100 µL of each test bacterial culture (10^9^ cells/mL), 2 mL soft agar (0.6% agar, 0.5% NaCl, 5 mM histidine, and 50 mM biotin, pH 7.4, 40–50 °C), 0.5 mL S9 mixture (if necessary), and AzP were mixed well in a test tube. Immediately following mixing, the sample was poured into a minimal agar plate (1.5% agar, Vogel-Bonner E medium containing 2% glucose), and the plate was incubated for 48 h at 37 °C in the dark. The revertant colonies were counted, and a positive mutagenic response was identified when (i) the number of revertants was at least double the spontaneous yield; (ii) a statistical significance (*p* ≤ 0.05) was found; and (iii) a reproducible positive dose response was present.

### 4.10. Sulforhodamine B (SRB) Assay 

The cell proliferations of A549 and Met-5A cells were determined using the SRB assay. Cells (2 × 10^4^) were dispensed in a 96-well plate for 24 h. Before being treated with varying concentrations of AzP (5–50 μM) or SAHA (5–50 μM) for 48 h, cells were fixed in 10% trichloroacetic acid for 10 min and washed with distilled water twice to remove excess trichloroacetic acid. Cells were stained with a 0.4% SRB solution dissolved in 1% acetic acid for 10 min and washed with 1% acetic acid twice to remove excess dye. The protein-bound dye was dissolved in 100 μL Tris-based solution (10 mM), and the absorbance was measured at 515 nm using a microplate reader. Cell Proliferation % = {(T48 − T0)/T0} × 100. Wherein, T48 represents the signal measured after 48-h treatment, T0 represent the signal measured before treatment at time 0. Relative cell proliferation (%) were expressed as mean ± SD compare with control. 

### 4.11. Chromosomal Aberrations

Chinese hamster ovary epithelial cells (CHO-K1, American Type Culture Collection, Manassas, VA, USA) were cultured in 6-cm dishes at a density of 2 × 10^5^ cells/mL for chromosomal abnormality analysis. The cells were treated with distilled water, mitomycin C (1 μg/mL), benzo(a)pyrene (5 μg/mL), or AzP (0.625, 1.25, or 2.5 mg/mL) with or without S9 metabolic activation. Colemid (0.1 μg/mL) was administered 3 h before trypsinization, followed by treatment with 0.9% sodium citrate at 37 °C and Carnoy’s solution (methanol:acetic acid, 3:1). The cells were stained with 3% Giemsa solution in 0.07 M phosphate buffer (pH 6.8) on glass slides. One hundred well-spread cells were observed for chromosomal alterations on each slide. The data were expressed as the total mean number of chromosomal aberrations per treatment ± standard deviation (SD) from three independent experiments.

### 4.12. Micronucleus Test 

Male ICR mice (*n* = 5) were administered AzP orally by gavage, at doses of 5, 50, and 500 mg/kg body weight. Cyclophosphamide (200 mg/kg body weight) was administered as a positive control through intraperitoneal injection. At 24, 48, and 72 h after dosing, 2-μL blood samples were collected from the tail vein onto a slide stained with acridine orange (40 μg/mL). The ratio (%) of polychromatic erythrocytes (PCEs) to total erythrocytes was determined using fluorescence microscopy. In addition, the frequency of micronucleated polychromatic erythrocytes (MNPCEs; ‰) was counted to a total of 1000 erythrocytes or PCEs per animal.

### 4.13. Statistical Analysis

All collected data were expressed as the means ± SD from at least 3 independent experiments (*n* ≥ 3). Statistically significant differences between groups were determined using one-way analysis of variance (ANOVA) where a *p* value < 0.05 was considered statistically significant.

## 5. Conclusions

In this study, we evaluated the efficacy of azatyrosine-phenylbutyric hydroxamide (AzP) on chemotherapy-induced gastrointestinal mucositis. Results showed that AzP has promise in the prevention of the gastrointestinal mucositis that occurs during chemotherapy, and shows no genotoxicity and no clastogenicity.

## Figures and Tables

**Table 1:** Pan histone deacetylase (HDAC) enzyme activity, subtype HDAC1 activity, subtype HDAC3 activity and subtype HDAC4 activity treated with azatyrosine-phenylbutyric hydroxamides (AzP) and suberoylanilide hydroxamic acid (SAHA).

Activity (IC_50_, nM)	AzP	SAHA	Ration: AzP/SAHA
Pan-HDAC	1650 ± 103	84.5 ± 13.7	20:1
HDAC1	3440 ± 680	566.5 ± 65.6	6:1
HDAC3	660 ± 99	72.5 ± 5.4	9.2:1
HDAC4	1970 ± 110	106.2 ± 27.3	106:1

**Table 2:** Genotoxicity evaluation of AzP.

Test Compound	Gene Mutation in Bacteria-Salmonella/Microsome Reversion Assay (Ames Test) of AzP									
AzP	Revertant colonies per plate (-S9)					Revertant colonies per plate (|S9)				
	TA98	TA100	TA102	TA1535	TA1537	TA98	TA100	TA102	TA1535	TA1537
	Negative					Negative				
	**Chromosome aberration assay of AzP in Chinese Hamster Ovary cells**									
	3 h without S9 metabolic activation			3 h with S9 metabolic activation				24 h without S9 metabolic activation		
	Negative			Negative				Negative		
	**Micronucleus Test of AzP on male ICR mice**									
	AzP treatment after 24 h			AzP treatment after 48 h				AzP treatment after 72 h		
	Negative			Negative				Negative		

## Supplementary Materials

The following are available online at http://www.mdpi.com/1422-0067/20/2/249/s1, Figure S1: Heat maps from hierarchical clustering analysis of the IEC-6 cells with different treatments (1% DMSO, LPS 500 ngml and/or AzP 20 microM) and detailed individual genes., Table S1: Gene Mutation in Bacteria- Salmonella/Microsome Reversion Assay (Ames test) of AzP, Table S2: Chromosome aberration assay of AzP in Chinese Hamster Ovary cells, Table S3: Micronucleus Test of AzP on male ICR mice.
