# Supplementary figures and images for: Efficacy of Azatyrosine-Phenylbutyric Hydroxamides, a Histone Deacetylase Inhibitor, on Chemotherapy-Induced Gastrointestinal Mucositis
Source: Int J Mol Sci. 2019 Jan 10;20(2):249. doi: 10.3390/ijms20020249 (PMC6359543; doi:10.3390/ijms20020249)

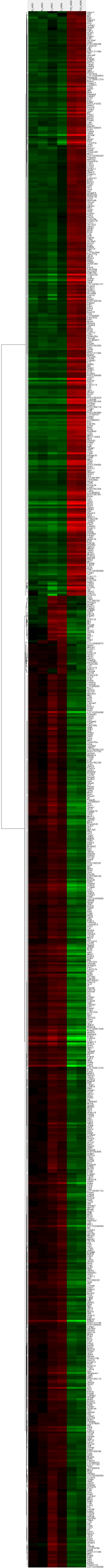

Supplement: Supplementary file 1 [file ijms-20-00249-s001.zip › Supplementary Fig 1. Heat maps from hierarchical clustering analysis of the IEC-6 cells with different treatments (1% DMSO, LPS 500 ngml andor AzP 20 microM) and detailed individual genes..gif]
