# Supplementary material for: Efficacy of Azatyrosine-Phenylbutyric Hydroxamides, a Histone Deacetylase Inhibitor, on Chemotherapy-Induced Gastrointestinal Mucositis
Source: Int J Mol Sci. 2019 Jan 10;20(2):249. doi: 10.3390/ijms20020249 (PMC6359543; doi:10.3390/ijms20020249)
Supplement: Supplementary file 1 [file ijms-20-00249-s001.zip › Supplementary Table 3.doc]

**Supplementary Table 3. Micronucleus Test of AzP on male ICR mice**

|  | Negative | AzP (mg/kg b.w.) | | | Positive |
| --- | --- | --- | --- | --- | --- |
|  | HPβCD | 5 | 50 | 500 | Cyclophosphamide 200 mg/kg |
| Number of Micronuclei (‰) | | | | | |
| Day 1 | 0.53±0.38 | 0.67±0.24 | 0.60±0.16 | 0.53±0.13 | 4.73±037*** |
| Day 2 | 0.33±0.00 | 0.40±0.16 | 0.67±0.24 | 0.47±0.17 | 4.80±0.08*** |
| Day 3 | 0.47±0.17 | 0.53±0.17 | 0.60±0.12 | 0.67±0.15 | 4.40±0.19*** |
| Reticuloctes(%) | | | | | |
| Day 1 | 5.93±0.32 | 6.07±0.44 | 6.33±0.24 | 5.73±0.24 | 1.60±0.12*** |
| Day 2 | 5.87±0.25 | 7.27±0.54 | 6.73±0.34 | 6.80±0.76 | 0.93±0.07*** |
| Day 3 | 6.07±0.34 | 5.80±0.34 | 6.73±0.51 | 6.20±0.50 | 1.07±0.16*** |

No significant change of micronuclei or Reticuloctes was shown under the AzP treatment groups. The data are expressed as the Mean ± SD. (N=5). Significant difference as ^*^: *p*<0.05, ^**^*p* < 0.01, ^***^*p* < 0.001 was compared with control and treatment group by one-way Anova.
