# Supplementary material for: Efficacy of Azatyrosine-Phenylbutyric Hydroxamides, a Histone Deacetylase Inhibitor, on Chemotherapy-Induced Gastrointestinal Mucositis
Source: Int J Mol Sci. 2019 Jan 10;20(2):249. doi: 10.3390/ijms20020249 (PMC6359543; doi:10.3390/ijms20020249)
Supplement: Supplementary file 1 [file ijms-20-00249-s001.zip › Supplementary Table 2.doc]

|  | Aberrant cell (%)^4^ | | Number of cells with structural aberrations (%)^3^ | | | | | |
| --- | --- | --- | --- | --- | --- | --- | --- | --- |
|  | With gap | Without gap | G | B | D | g | b | e |
|  | 3 hours without S9 metabolic activation | | | | | | | |
| Negative^1^ | 1.67±0.58 | 0.00±0.00 | 0.00±0.00 | 0.00±0.00 | 0.00±0.00 | 1.67±0.58 | 0.00±0.00 | 0.00±0.00 |
| Positive ^2^ | 7.67±1.15*** | 4.00±1.00*** | 0.00±0.00 | 0.00±0.00 | 1.67±0.58 | 3.67±0.58* | 0.33±0.58 | 2.00±1.00** |
| AzP (μg/mL) | | | | | | | | |
| 5 | 2.33±0.58 | 0.67±0.58 | 0.33±0.58 | 0.00±0.00 | 0.67±0.58 | 1.33±0.58 | 0.00±0.00 | 0.00±0.00 |
| 10 | 1.67±0.58 | 0.33±0.58 | 0.00±0.00 | 0.00±0.00 | 0.33±0.58 | 1.33±0.58 | 0.00±0.00 | 0.00±0.00 |
| 20 | 3.33±0.58 | 1.00±1.00 | 0.00±0.00 | 0.00±0.00 | 1.00±1.00 | 2.33±0.58 | 0.00±0.00 | 0.00±0.00 |
|  | 3 hours with S9 metabolic activation | | | | | | | |
| Negative^1^ | 2.00±0.00 | 0.33±0.58 | 0.00±0.00 | 0.00±0.00 | 0.33±0.58 | 1.67±0.58 | 0.00±0.00 | 0.00±0.00 |
| Positive ^2^ | 8.33±3.21** | 7.00±3.6** | 0.00±0.00 | 0.00±0.00 | 1.00±0.00 | 1.33±0.58 | 2.33±0.58*** | 3.67±2.52* |
| AzP (μg/mL) | | | | | | | | |
| 5 | 2.00±0.00 | 0.33±0.58 | 0.00±0.00 | 0.00±0.00 | 0.00±0.00 | 1.67±0.58 | 0.33±0.58 | 0.00±0.00 |
| 10 | 2.00±1.00 | 0.00±0.00 | 0.00±0.00 | 0.00±0.00 | 0.00±0.00 | 2.00±1.00 | 0.00±0.00 | 0.00±0.00 |
| 20 | 1.33±0.58 | 0.33±0.58 | 0.00±0.00 | 0.00±0.00 | 0.00±0.00 | 1.00±0.00 | 0.33±0.58 | 0.33±0.58 |
|  | 24 hours without S9 metabolic activation | | | | | | | |
| Negative^1^ | 3.67±0.58 | 2.67±1.55 | 0.00±0.00 | 0.00±0.00 | 1.67±1.15 | 1.00±1.00 | 0.67±0.58 | 0.33±0.58 |
| Positive ^2^ | 10.00±1.73*** | 4.00±3.61 | 0.00±0.00 | 0.00±0.00 | 0.33±0.58 | 2.67±1.53 | 1.33±1.15 | 4.33±2.52* |
| AzP (μg/mL) | | | | | | | | |
| 5 | 4.33±0.58 | 1.67±1.53 | 0.33±0.58 | 0.00±0.00 | 0.33±0.58 | 2.33±1.53 | 1.00±1.00 | 0.33±0.58 |
| 10 | 4.00±1.00 | 2.67±1.53 | 0.67±0.58 | 0.00±0.00 | 0.67±0.58 | 1.33±0.58 | 1.33±0.58 | 0.67±0.58 |
| 20 | 4.33±0.58 | 0.67±0.58 | 0.33±0.58 | 0.33±0.58 | 0.00±0.00 | 3.33±0.58 | 0.00±0.00 | 0.00±0.00 |

**Supplementary Table 2. Chromosome aberration assay of AzP in Chinese Hamster Ovary cells**

No significant aberration was shown under the treatment groups. The data are expressed as the Mean ± SD. (N=3). Significant difference as ^*^: *p*<0.05, ^**^*p* < 0.01, ^***^*p* < 0.001 was compared with control and treatment group by one-way Anova.

^1^ Solvent (distilled water) was used as negative control.

^2^ Mitomycin C 1μg/mL was used as positive control in without S9 metabolic activation condition; benzo(a)pyrene 5μg/mL was used as positive control in with S9 metabolic activation condition.

^3^ G: chromosome gap; B: chromosome break; D: dicentric; g: chromatid gap; b: chromatid break; e: exchange.

^4^ Chromatid and chromosome gaps were recorded but separated into two groups, with gaps and without gaps.
