# Supplementary material for: Efficacy of Azatyrosine-Phenylbutyric Hydroxamides, a Histone Deacetylase Inhibitor, on Chemotherapy-Induced Gastrointestinal Mucositis
Source: Int J Mol Sci. 2019 Jan 10;20(2):249. doi: 10.3390/ijms20020249 (PMC6359543; doi:10.3390/ijms20020249)
Supplement: Supplementary file 1 [file ijms-20-00249-s001.zip › Supplementary Table 1.doc]

**Supplementary Table 1. Gene Mutation in Bacteria- Salmonella/Microsome Reversion Assay (Ames test) of AzP**

| Test Compound | Dose  (mg/plate) | Revertant colonies per plate (-S9)  (Mean±SD) | | | | | Revertant colonies per plate (+S9)  (Mean±SD) | | | | |
| --- | --- | --- | --- | --- | --- | --- | --- | --- | --- | --- | --- |
|  |  | TA98 | TA100 | TA102 | TA1535 | TA1537 | TA98 | TA100 | TA102 | TA1535 | TA1537 |
| AzP | 0.00245 | 42±4.6 | 81±5 | 141±4.4 |  | 15±0.5 | 59.7±3.8 | 106.7±2.2 | 219.7±6.9 |  | 32.3±3 |
|  | 0.0049 | 29±5.7 | 78.7±2.7 | 142.3±4.7 |  | 10.7±0.9 | 58.3±4.7 | 106±4.4 | 225.7±4.5 |  | 35.3±2.2 |
|  | 0.0098 | 24±5.4 | 81.7±2.7 | 140.3±7.9 |  | 10±0.6 | 56.3±3.4 | 107.3±1.3 | 215±4 |  | 29.3±2.2 |
|  | 0.0195 | 33.7±3 | 89.7±9 | 139.5±5.6 | 52±1.2 | 10±2 | 59.7±0.7 | 110.3±3.8 | 230.7±2 |  | 27.3±2.7 |
|  | 0.0391 | 33.7±2.8 | 90.8±9.6 | 138.3±8.5 | 47.3±9 | 11.8±6 | 62.7±2.3 | 150.3±19.7 | 212.2±9.2 | 46.5±3.3 | 22.1±2.5 |
|  | 0.0781 | P | 88.7±1.2 | 152.7±11.3 | 59±0.6 | 12±4.9 | 56.3±2.2 | 153±3.1 | 233.3±9.8 | 50.5±4.9 | 16±2.5 |
|  | 0.156 |  | P | P | 61.3±2.2 | 14.7±2 | 61±1.2 | 104±8 | 217.3±4.8 | 52.5±5.2 | 18±1 |
|  | 0.3125 |  |  |  | 54.6±7.2 | P | 69.7±5.8 | 92.2±5.2 | 230.1±8.4 | 51.7±3.6 | P |
|  | 0.625 |  |  |  | 52.2±13.4 |  | 64±2.9 | P | 217.4±7.3 | 59.2±3.3 |  |
|  | 1.25 |  |  |  | P |  | P |  | P | 67±3.2 |  |
|  | 2.5 |  |  |  |  |  |  |  |  | 58±2.1 |  |
|  | 5 |  |  |  |  |  |  |  |  | P |  |
|  | | | | | | | | | | | |
| Positive  Control |  | 1549.3±37.6 | 1433.6±94.4 | 1160±23.1 | 1506.7±62.3 | 1671.8±184 | 1632±27.5 | 1514.7±6.9 | 1409.8±78.2 | 1182.9±81.6 | 1282.8±109 |
| Control |  | 36.3±1.5 | 76.2±1 | 147.17±1.2 | 45.6±3.8 | 11.5±1.2 | 53.3±8.8 | 122.8±12.8 | 224.8±4.1 | 47±2.4 | 29±2.9 |

Data were presented as Mean±S.D (N≥3). No significant changes of revertants were observed in five strains with treating Memoregain Capsule. * was marked when the number of revertants was twice than control at TA98, TA100 and TA102, and triple than control at TA1537, TA1535.

^1^ Solvent (distilled water) was used as negative control.

^2^Positive control in –S9 plate: 9-Aminoacridine 50 μg /plate (TA1537); 4-Nitro-o-phenylenediamine 2.5 μg / plate (TA 98); Sodium Azide 5 μg / plate (TA 100 and TA 1535); Mitomycin C 0.5 μg / plate (TA 102). Positive control in +S9 plate: 2-aminoanthrene 5 μg /plate (TA 1537, TA100, TA102 and TA1535); Benzo(a)pyrene [B(a)p] 5 μg /plate (TA 98).
